# Effects of statins on the secretion of human serum albumin in cultured HepG2 cells

**DOI:** 10.1186/1423-0127-16-32

**Published:** 2009-03-16

**Authors:** Chung-Eun Ha, Ji-Sook Ha, Andre G Theriault, Nadhipuram V Bhagavan

**Affiliations:** 1 Department of Native Hawaiian Health, John A. Burns School of Medicine, University of Hawaii at Manoa, 651 Ilalo Street, Honolulu, HI 96813, USA; 2 Department of Anatomy, Biochemistry, & Physiology, John A. Burns School of Medicine, University of Hawaii at Manoa, 1960 East-West Road, Honolulu, HI 96822, USA; 3 Department of Medical Technology, John A. Burns School of Medicine, University of Hawaii at Manoa, 1960 East-West Road, Honolulu, HI 96822, USA

## Abstract

Statins reduce cholesterol biosynthesis by inhibiting HMG-CoA reductase and thereby lower total cholesterol and LDL cholesterol levels in serum, which in turn lower the incidence of cardiovascular disease (CVD). Statins are also known to modulate various cellular functions such as gene expression, cell proliferation, and programmed cell death through inhibition of downstream intermediates in cholesterol synthesis. In this study, we have investigated the possible effects of statins on the secretion of serum albumin from cultured HepG2 cells since high levels of serum albumin are associated with reduced risks for CVD and statins are effective in lowering the risk of CVD through other effects in addition to their effects on serum total cholesterol and LDL cholesterol levels, known as pleiotropic effects. Our results showed that simvastatin increased HSA secretion up to 32.3% compared to the control group. Among 3 statin analogs we tested, simvastatin exhibited the highest stimulatory effects on HSA secretion compared to the control group. Our study also showed that the increased HSA secretions from HepG2 cells by simvastatin treatments were due to the increased rate of HSA synthesis, not due to the reduced posttranslational degradation rate of HSA. Our finding suggests another added benefit of statins' treatments in preventing CVD through stimulation of HSA biosynthesis.

## Background

Statins are one of the most widely prescribed medications to patients in the high-risk group of developing CVDs to lower total serum and LDL cholesterol levels. Statins inhibit 3-hydroxy-3-methylglutaryl coenzyme A (HMG-CoA) reductase in the cholesterol biosynthesis, thereby lowering serum LDL cholesterol production, up-regulating the synthesis of LDL receptors in the liver, and in turn resulting in decreased levels of circulating total cholesterols[1]. In addition to their major effects on serum LDL cholesterol, recent studies have shown that statins also exert wide variety of other effects on cellular metabolism. These other effects of statins, known as pleiotropic effects include modulation of gene expression, reduction of endothelial dysfunction, inhibition of inflammatory reactions, antioxidant effects, inhibition of smooth muscle proliferation, and induction of apoptosis [2-9]. These findings suggest that the overall cardioprotective effects of statin treatments resulted not only from the decreased serum LDL cholesterol level but also from statins' potential pleiotropic effects. In fact, several studies have suggested that statins' pleiotropic effects may provide greater protection against pathogenesis of CVDs than their cholesterol lowering effects do [10,11].

Human serum albumin (HSA) is the principal carrier of unesterified free fatty acids in serum and its major functions include maintaining osmotic pressure, transporting endogenous and exogenous ligands. Many epidemiological studies have shown that high plasma levels of HSA are also associated with reduced risks for CVD [12-19]. Our previous study has shown that HSA participates in the cholesterol efflux from cultured endothelial cells and due to its high concentration (40 g/L) in plasma, HSA may contribute significantly to cholesterol efflux together with HDL mediated cholesterol efflux [20]. Other contribution of HSA's anti-atherogenic effects might be due to its anti-oxidant properties. HSA serves as an important antioxidant in the plasma because of its free cysteine residue at amino acid position 34 [21]. Previous study has shown that the occurrence of oxidized LDLs (ox-LDLs) can be attributed to the reduced synthesis of HSA that is often observed in hypercholesterolemia and diabetes patients [22]. Therefore, antioxidant capacity of the body fluids may significantly be affected by changes in the concentration of serum albumin. Many of HSA's anti-atherogenic functions overlap with statins' cardioprotective effects in the body. Therefore, in the present study we investigated whether statin's effects include the modulation of albumin synthesis and secretion from the cultured HepG2 cells since previous studies have shown that statins effects in cellular level include the modulation of certain gene expression.

The structure of HMG-CoA reductase and complexes with six statins have been determined by X-ray crystallographic studies [23]. All statins function similarly by binding to the active site of HMG-CoA reductase and thus inhibiting the enzyme reaction. However, the different structures and binding characteristics of statins are responsible for the differences in potency of HMG-Co A reductase inhibition and other variations in pharmacologic properties [23-25]. Since all of the statins inhibit HMG-CoA reductase with minor differences in their effects, their pleiotropic effects might also be different among statins. In the present study, we compared the effects of three statin analogs, simvastatin, lovastatin, and pravastatin on HSA secretion from HepG2 cells.

## Materials and methods

### Materials

Human hepatoma HepG2 cells (HB8065) were obtained from American Type Culture Collection (Rockville, MD). Cell culture media (RPMI 1640) and fetal bovine serum (FBS) were obtained from Invitrogen Life Technologies (Grand Island, NY). Culture dishes were obtained from Corning Costar (Lowell, MA). Prestained SDS-PAGE Standard, Affi-Gel Protein A was from Bio-Rad (Hercules, CA). Simvastatin, lovastatin, pravastatin and anti-Human albumin antibody were from Sigma (St. Louis, MO). [^35^S] protein-labeling mix was from Perkin Elmer Life Science Research Products (Boston, MA). ENHANCE™ and autoradiography films were purchased from NEN Life Science Research Products (Boston, MA). RNeasy Mini Kit and Onestep RT-PCR kit were obtained from Qiagen (Valencia, CA). Human Albumin ELISA Quantitation Kit was obtained Bethyl Inc. (Montgomery, TX). Oligonucleotide primers for RT-PCR for HSA and GAPDH genes were from Midland Certified Reagent (Midland, TX).

### Cell Culture

HepG2 cells were maintained in RPMI medium supplemented with 10% fetal bovine serum (FBS), 1% glycine, and 1% antibiotics at 37°C as previously described [26]. Cell cultures were allowed to reach 90% confluency. The cells were then treated with appropriate culture media containing 100 nM to 10 μM of simvastatin, lovastatin, and pravastatin and incubated at 37°C for 24 hours.

### Secretion of HSA measured by ELISA

The amount of HSA secreted into the culture medium was determined by Human Albumin ELISA Kit. Spectrophotometric measurements were made by using a Microplate reader (VERSA_max_, Molecular Devices, CA). Cells treated with or without statins were harvested and lysed in 0.5 ml of 0.1 N NaOH solution. The total cellular protein contents were measured by bicinchoninic acid (BCA) Protein Assay Kit (Pierce Chemical, Rockford, IL).

### HSA synthesis measured by incorporation of [^35^S]

The effects of simvastatin on the synthesis/secretion of HSA in HepG2 cells were studied by measuring the amount of [^35^S] radiolabeled methionine incorporated into HSA, which was secreted into the culture medium. HepG2 cells were incubated in methionine/cysteine free RPMI medium containing simvastatin and [^35^S] methionine/cysteine (100 μCi/ml) for 20 minute. After labeling, the radioactivity was chased for 1 h and then, the culture medium and cell lysates were subjected to the measurement of HSA concentration.

### Pulse-Chase Analysis of HSA Synthesis and Secretion

To determine whether effects of statins on HSA secretion were due to altered synthesis rate or due to changed posttranslational degradation rate, the amount of HSA *de novo *synthesis was measured by a modified pulse-chase method as previously described by Theriault et al. [27]. To determine the amount of *de novo *HSA biosynthesis, cells were preincubated in methionine and cysteine free RPMI media with or without statins for 30 minutes. After preincubation, cells were pulsed with a [^35^S] methionine/cysteine-labeling medium supplemented with 100 μCi/ml [^35^S] methionine/cysteine (Perkin Elmer, Waltham, MA), in methionine and cysteine-free RPMI for 20 minutes. After the pulse treatment, the cells were washed with PBS two times and placed in the chase medium containing 1000-fold excess unlabeled methionine/cysteine for 20 & 120 minutes in the presence of various statins. At each chase time, the cells were harvested and lysed in PBS buffer containing 1% NP40, 1% deoxycholate, 5 mmole/L EDTA, and 1 mmole/L EGTA. The lysates were centrifuged for 10 minutes in a microcentrifuge at 10,000 rpm for immunoprecipitation and analyzed by SDS-PAGE and fluorography as described previously [27].

### Measurement of HSA mRNA expression

To determine mRNA synthesis levels in the cells, total RNA was extracted by RNeasy Kit (Qiagen, CA) and was subjected to RT-PCR reactions by using a commercial Qiagen Onestep RT-PCR Kit (Qiagen, CA). The glyceraldehyde 3-phosphate dehydrogenase (GAPDH) was used as a control for RT-PCR reactions. The RT-PCR products of HSA and GAPDH mRNA were analyzed on 2% agarose gel electrophoresis using ethidium bromide staining. DNA band intensities were determined by Gel Documentation System (Bio-Rad Laboratories, Hercules, CA). Using the amount of GAPDH mRNA amplified as control, the mRNA levels of HSA were normalized and compared.

### Statistical analysis

All data were expressed as the mean ± SD of at least three experiments performed in duplicate. Differences between group means were analyzed by using Student t-tests.

## Results

### Determination of optimal simvastatin concentration for the study of statins' effects on HepG2 cells

**Figure 1:**
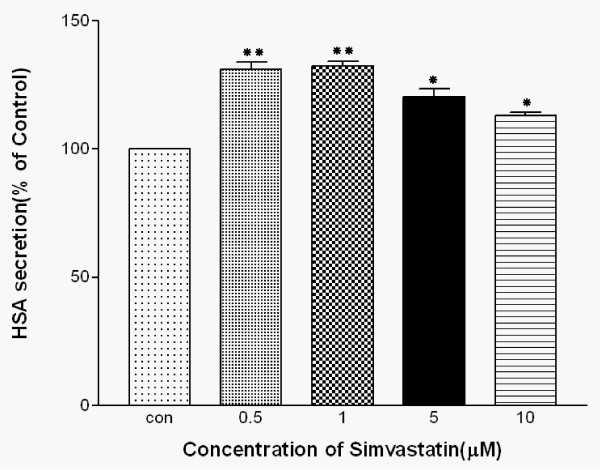
**Effects of simvastatin on HSA secretion from HepG2 cells**. Cells were exposed to various concentrations (0, 0.5, 1, 5, 10 μM) of simvastatin for 24 hours. HSA secretion was measured by ELISA. Data presented are mean ± SD from three independent experiments performed in triplicate. *P < 0.05, **P < 0.01 vs. without simvastatin.

To determine the optimal conditions for the analysis of statins' effects on cell culture, HepG2 cells were incubated with various concentrations of simvastatin (0, 0.5, 1.0, 5, 10 μM) for 24 hours. The treatments of 0.5, 1.0, 5, and 10 μM simvastatin increased HSA secretion up to 31.2, 32.3, 20.3, and 13.1%, respectively compared to control group cells without simvastatin treatments. The maximum effect on HSA secretion was observed with 1 μM simvastatin added to the culture medium (Fig. 1). Further increases in the concentration of simvastatin did not enhance its stimulatory effect on HSA secretion from HepG2 cells. Therefore, we used 1 μM and 5 μM simvastatin concentrations for our study. Also, incubation time of 24 hours did not alter either the morphology or viability of HepG2 cells.

### Effects of simvastatin, lovastatin and pravastatin on HSA secretion

**Figure 2:**
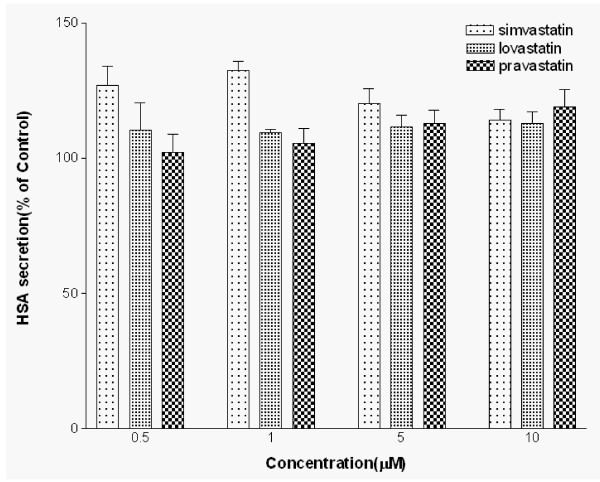
**Effects of simvastatin, lovastatin and pravastatin on HSA secretion**. Cells were exposed to different concentrations (0, 0.5, 1, 5, 10 μM) of simvastatin, lovastatin and pravastatin for 24 hours. HSA secretion was measured by ELISA. Data are mean ± SD from three independent experiments performed in triplicate.

The stimulatory effect of simvastatin on HSA secretion from HepG2 cells was compared with those of lovastatin and pravastatin. As seen in Fig. 2, three statin analogs showed different effects on HSA secretion in dose dependent experiments. Lovastatin had the minimal effects and pravastatin increased HSA secretion only at high concentrations (13%, 5 μM; 19%, 10 μM) (Fig 2). Among these statin analogs, simvastatin showed the highest increase in HSA secretion and was chosen for the further studies.

### Time-dependent effect of simvastatin on HSA secretion

**Figure 3:**
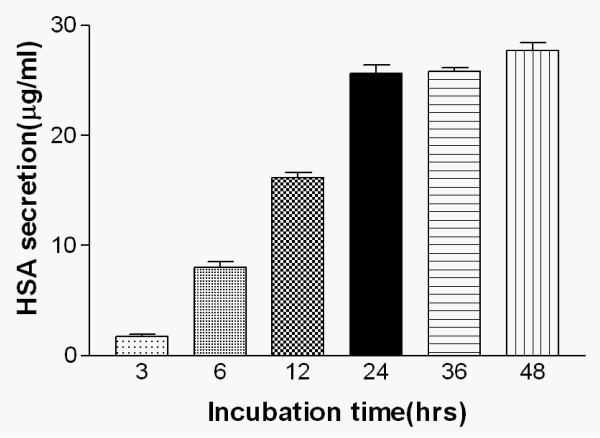
**Time-dependent effect of simvastatin on HSA secretion in HepG2 cells**. Cells were treated with 1 μM of simvastatin for 3, 6, 12, 24, and 48 hours and then HSA was measured by ELISA. Values represent the mean ± SD from three independent experiments performed in triplicate.

To determine the optimal incubation time for our study, we tested statins' effects on HSA secretion with different incubation times (3, 6, 12, 24, 36, and 48 hours). Among these different time settings, statins' effects on HSA secretion reached the maximum rate at 24 hours of incubation time. Therefore, for all further experiments we used 24-hour incubation time (Fig. 3).

### Simvastatin increases synthesis and secretion of HSA in HepG2 cells

**Figure 4:**
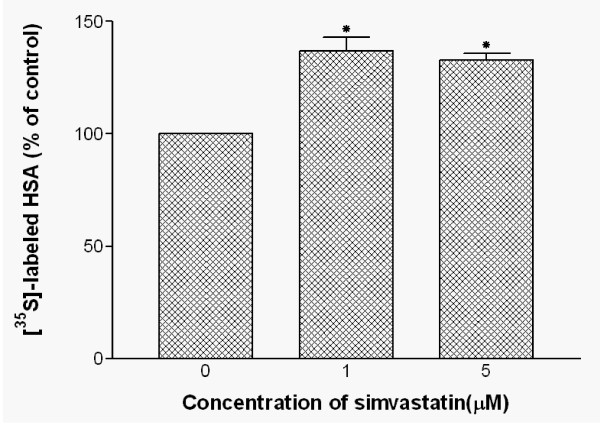
**Effects of simvastatin treatments on intracellular HSA synthesis in HepG2 cells**. HepG2 cells were treated with 0, 1, and 5 μM simvastatin for 22 hours and further incubated with [^35^S]-methionine. After incubation, cells were lysed with lysis buffer and the resulting cell lysates were subjected to immunoprecipitation. Cell lystaes were further analyzed by SDS-PAGE/Fluorography and radioactivity of HSA protein bands were quantified by liquid scintillation counting. Data are mean ± SD from three independent experiments performed in duplicate. *P < 0.05 vs. without simvastatin.

The effect of simvastatin on *de novo *synthesis of HSA was determined by incubating cells with [^35^S] methionine for 22 hours in the presence of 1 or 5 μM of simvastatin. *De novo *synthesis of HSA was determined by immunoprecipitation and radioactivity measurement. As shown in Fig 4, the level of HSA synthesis in the presence or absence of 1 and 5 μM of simvastatin was affected. There was a significant increase in the intracellular levels of HSA. In the presence of 1 and 5 μM simvastatin, *de novo *synthesis of HSA was increased by 37% and 33%, respectively.

**Figure 5:**
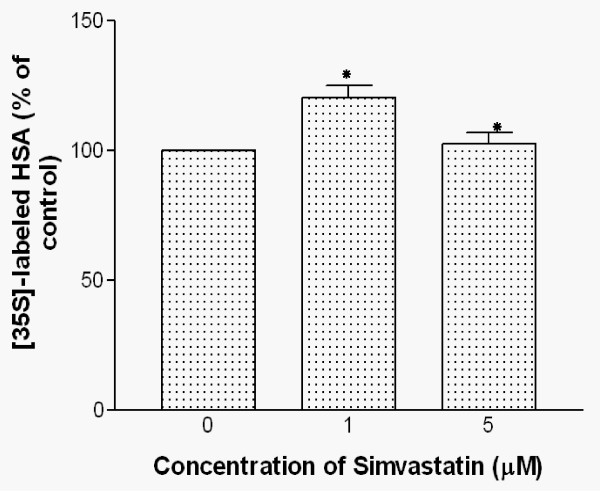
**Effects of simvastatin treatment on HSA secretion into culture media from HepG2 cells**. HepG2 cells were treated with 0, 1, and 5 μM simvastatin for 22 hours in RPMI media. Cells were incubated additional 2 hours with [^35^S]-methionine/cysteine. Media was collected and the secreted HSA in the media was immunoprecipitated with a specific anti-HSA antibody followed by SDS-PAGE. Radioactive quantification of HSA bands was performed by liquid scintillation counting. Data are mean ± SD from three independent experiments performed in triplicate. *P < 0.05 vs. without simvastatin.

Measurement of HSA secreted into the culture media after 2 hours of incubation with simvastatin in HepG2 cells showed that pretreatment with 1 μM simvastatin induced a smaller but significant stimulation (~20%) of HSA secretion. However, treatment of 5 μM simvastatin had no apparent effects on HSA secretion when compared to the treatment of 1 μM simvastatin as shown in Fig. 5. The data suggest that treatment of 1 μM simvastatin induced the increases in both *de novo *synthesis and secretion of HSA into the medium. However, 5 μM simvastatin treatment increased *de novo *synthesis of HSA but not HSA secretion into the medium.

### Simvastatin effects on total protein synthesis

**Figure 6:**
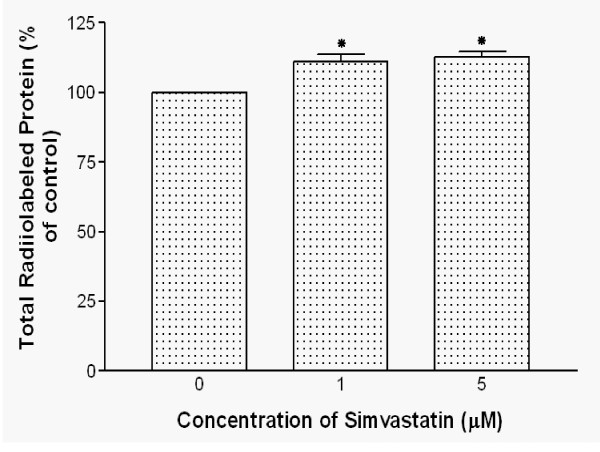
**Effects of simvastatin on total radiolabeled protein in HepG2 cells**. Confluent HepG2 cells were treated with 1 and 5 μM simvastatin for 22 hours and then labeled for 2 hours with [^35^S]-methionine/cysteine. Total protein was determined by TCA precipitation. Data are mean ± SD from three independent experiments performed in triplicate. *P < 0.05 vs. without simvastatin.

To determine whether simvastatin specifically increased *de novo *synthesis of HSA, total protein synthesis of HepG2 cells was examined after 2-hour incubation with [^35^S] methionine/cysteine by TCA precipitation. The cells were treated with 1 μM and 5 μM of simvastatin for 22 hours. The results showed that there was 13% increase in total protein synthesis in simvastatin treated cells compared to the control group. However, different amounts of simvastatin treatment (1 μM and 5 μM) did not show differences in total protein synthesis change (Fig. 6).

### Simvastatin effects on intracellular turnover rate of HSA

A modified pulse-chase labeling experiment was performed to determine whether the effect of simvastatin on HepG2 cells was due to the increased synthesis of HSA or the decreased post-translational degradation level of HSA. The result showed that incorporation rates of [^35^S]-methionine into HSA during the pulse was higher in cells preincubated with simvastatin than control cells preincubated without simvastatin. After 2 hours of chase incubation, HSA secretion into the medium was determined and the results showed that there were no significant changes in HSA degradation in the cells treated with or without 1 μM simvastatin. However, the cells treated with 5 μM of simvastatin induced the increased degradation of HSA compared to the cells without simvastatin treatment (Table 1). The increased HSA degradation rate by 5 μM simvastatin treatment explains that HSA secretion from the cells treated with 5 μM simvastatin was not changed significantly compared to the control cells without simvastatin treatment. Simvastatin treatment to the cultured HepG2 cells lead to the increased synthesis of HSA and at higher concentration the treatment induced both HSA synthesis increase and degradation.

**Table 1:** Effects of simvastatin on intracellular HSA degradation in HepG2 cells.

	HSA (10^2 ^× dpm)		
	Control	Simvastatin (1 μM)	Simvastatin (5 μM)

Intra peak *	350 ± 31	414 ± 24	403 ± 52

Depletion	216	237	243

Secretion	168	173	143

Degradation	48	64	100

% Recovery **	86.3	84.5	75.2

Cells were incubated for 22 hours with 0, 1, and 5 μM simvastatin, then cell were pulse-labeled for 20 minutes with [^35^S] methionine. After pulse-labeling, media were washed and incubated for 20, 120 minutes in media containing an excess of unlabeled methionine/cysteine. At the end of each chase period, solubilized cell lysates and media were subjected to immunoprecipitation. Quantification of labeled HSA was determined by SDS-PAGE and scintillation counting. Data presented are from three separate experiments done in duplicate.

* Intra peaks were calculated from the HSA content at 20 minutes of chase

** % recovery represents total labeled apoB (cell lysate + media) remained after 2-hour chasing.

### Simvastatin effects on HSA mRNA levels in HepG2 cells

**Figure 7:**
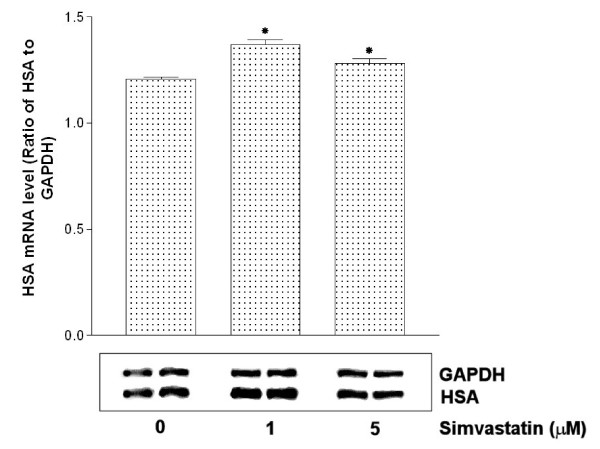
**Effects of simvastatin on HSA mRNA expression in HepG2 cells**. HepG2 cells were treated with 0, 1, 5 μM simvastatin for 22 hours. Total RNA was isolated and RT-PCR was subjected. HSA mRNA levels were quantified as described in Methods. Each band density of HSA was normalized by GAPDH and expressed as ratio of HSA mRNA to GAPDH mRNA. Bottom panels are representative of four independent experiments. Bar graph is mean ± SD from these experiments

To confirm the increased HSA synthesis in the presence of simvastatin, we used RT-PCR technique to measure the changes in the intracellular levels of HSA mRNA synthesis. The results indicated that the cells treated with 1 and 5 μM of simvastatin showed that HSA mRNA synthesis was increased by 13.6% and 6%, respectively compared to the level of HSA mRNA synthesis of control cells without treatment of simvastatin (Fig 7). This result further validates our findings that the effects of simvastatin on HSA secretion were mainly due to increased synthesis of HSA in the cells treated with simvastatin, not the results of reduced degradation of HSA.

## Discussion

Statins have been widely prescribed to treat hypercholesterolemia in an effort to reduce the risk of developing CVDs. A number of studies on statins' effects on cholesterol metabolism provided solid evidence that statins are very effective in lowering serum LDL cholesterol levels, thereby reducing the incidence of CVD cases. Many studies have shown that other effects of statins includes non-lipid related physiologic effects such as down-regulation of cell adhesion molecule expressions, angiotensin-converting enzyme (ACE) gene expression, endothelin-1 receptor expression and C-reactive protein expression [28-32]. These other effects of statins, so called pleiotropic effects also included up-regulation of certain genes such as collagen gene expression. These studies on statins' effects on various metabolic pathways suggested that statins not only served as major inhibitors for HMG-CoA reductase in cholesterol synthesis but also served as major metabolic modulators on the expression of various other metabolites, which participate in other distinctive processes in the body.

Albumin is the major plasma protein, which mainly responsible for maintaining oncotic pressure of plasma. Another main function of albumin in circulation includes transportation of major endogenous and exogenous metabolites and compounds such as thyroxine, bilirubin, free fatty acids, digoxin, ibuprofen. However, recent studies on albumin's other functions revealed that albumin plays an important role in the pathogenesis of cardiovascular diseases. Previous epidemiological studies have shown that the levels of HSA in the serum can be served as an independent risk factor for mortality and morbidity [17,33,34]. Also, it was shown that HSA possesses cardioprotective function and its high levels in serum were correlated with lowered incidence of CHD [19]. Furthermore, HSA participates in cholesterol efflux from peripheral tissues and contributes to the overall cholesterol efflux along with the contributions from HDL. In rodents, one study suggested that albumin contributes up to 24% of non-esterified cholesterol transport due to its high concentration in serum (35 – 50 grams/liter) [20,35]. Since many studies have shown that statins possess non-lipid pleiotropic effects on other metabolite expressions, we hypothesized that statins may also affect the level of HSA synthesis in the hepatocytes. In this study, we chose to investigate the effects of statins on HSA synthesis and secretion by using cultured HepG2 cells.

Our study showed that statins increased albumin secretion rate in cultured HepG2 cells by as much as 30%. We found that among three different analogs of statin, simvastatin showed the most significant effects on HSA secretion at concentrations ranges from 0.5 to 5 μM. These results indicate that the effect of statins on albumin secretion may complement to the effectiveness of statins' cholesterol lowering capacity. Furthermore, previous clinical studies showed that the mean reductions in total cholesterol in simvastatin treated group were highest (25%) compared to lovastatin treated group (17%) and pravastatin treated group (20%) [36]. The stimulation of albumin secretion by statins might be due to the inhibition of *Ras*-mediated signal transduction pathway in the regulation of hepatic cell growth by inhibiting mevalonate synthesis, thereby inhibiting posttranslational modification of *Ras *proteins. Statins are known to inhibit *Ras *farnesylation, resulting in the accumulation of inactive *Ras *proteins in the cytoplasm [37]. Previous study has shown that *Ras *proteins suppress albumin enhancer function possibly mediated through AP-1 interaction and therefore, inhibition of *Ras *proteins should result in increased expression of HSA synthesis [38].

The physiological processes between albumin synthesis and degradation maintain serum albumin concentration. Measurement of albumin synthesis rate should provide more dynamic insight into the effects of statin and its analogs on modulation of albumin metabolism than measurement of albumin degradation rate. To test this hypothesis, we used ELISA technique to measure secreted albumin and RT-PCR method to quantify albumin mRNA synthesis.

Our results showed that 1 μM simvastatin showed 30% increase in HSA secretion compared to control group and 13.6% increase in HSA mRNA synthesis. The 30% increase in HSA secretion from HepG2 cells incubated in the presence of 1 μM simvastatin could be accounted by 13.6% increase in HSA mRNA synthesis since one molecule of mRNA is used for the synthesis of many molecules of HSA. To validate our findings on simvastatin effects on HSA secretion, we determined total protein synthesis in the cultured HepG2 cells in the presence of 1 and 5 μM simvastatin and showed that 1 and 5 μM simvastatin increased 11% and 13% of total protein synthesis. These findings imply that simvastatin's effects on HSA secretion was not through increasing total protein synthesis of HepG2 cells, rather simvastatin's effects on HSA synthesis was specific process.

Various concentrations of pravastatin showed dose-dependent effects on HSA secretion whereas the effects of lovastatin showed dose-independent effects on albumin secretion. However, simvastatin has shown a biphasic effects in HSA secretions. The cells treated with 1 μM simvastatin showed better stimulation in HSA secretions (32.3%) compared to the cells treated with 5 μM simvastatin (13.1%). The differences in HSA secretions at two different simvastatin concentrations correspond to the different effects on HSA mRNA synthesis in HepG2 cells (Fig 7). This suggests that simvastatin's effects on HSA synthesis might be through regulation at the transcriptional level.

To further confirm the simvastatin's effects on HSA synthesis, we used [^35^S] incorporation technique to test the 30% increase in HSA secretion was the result of new HSA protein synthesis, not the result of reduced degradation of HSA in the cells. Our results clearly indicated that new synthesis of HSA was responsible for the increased HSA synthesis under the treatment of simvastatin. Also, studies on the degradation rate of HSA in the presence of simvastatin showed that there were no apparent changes in the intracellular degradation of HSA. In toto, our results specifically suggest that simvastatin induces increased HSA gene expression and results in increased HSA secretion from HepG2 cells. These findings add another beneficial effect of simvastatin treatment to the growing number of known benefits of HMG-CoA inhibitor treatments. Increased in HSA synthesis under the influence of simvastatin may provide synergic effects of simvastatin in lowering risk factors for developing CHD since previous epidemiologic studies have shown that high HSA levels are associated with reduced risk for these diseases. The mechanism of simvastatin effects on up-regulation of HSA synthesis needs further studies.

## Competing interests

The authors declare that they have no competing interests.

## Authors' contributions

CH participated in the overall planning of experiments, research design, data analysis & interpretation, and manuscript preparation. JH performed experimental studies of cell cultures, molecular biology, and pulse-chase experiments. AGT provided support in cell culture studies and participated in planning the pulse-chase experiments. NVB was involved in the generation of the conceptual aspects and overall design and also assisted in manuscript preparation. All authors read and approved the final manuscript.
